# Letter to Editor: “Integrating machine learning and molecular docking to decipher the molecular network of aflatoxin B1-induced hepatocellular carcinoma”

**DOI:** 10.1097/JS9.0000000000002855

**Published:** 2025-06-27

**Authors:** Xiong Teng, Chuang-Ye Han, Tao Peng

**Affiliations:** Department of Hepatobiliary Surgery, The First Affiliated Hospital of Guangxi Medical University, Nanning, People’s Republic of China

*Dear Editor*,

We read with great interest the recent article by Gao *et al*^[1]^ published in your esteemed journal, titled “*Integrating machine learning and molecular docking to decipher the molecular network of aflatoxin B1-induced hepatocellular carcinoma.*” The authors employed a combination of advanced computational methods to innovatively explore the molecular mechanisms of aflatoxin B1 (AFB1)-induced hepatocellular carcinoma (HCC). Their systematic integrative analysis strategy provides valuable insights into this complex disease process and is commendable.

However, for a more comprehensive assessment of the study’s findings and their potential applications, we believe several aspects warrant further consideration and discussion.

First, the study initially identified 48 candidate genes through gene set intersection, subsequently narrowing these down to 6 core genes using machine learning methods – an innovative multi-step screening strategy. When evaluating the intersection of AFB1-predicted targets and HCC-related genes, providing a statistical significance assessment of this overlap, for example by using a hypergeometric distribution test, would more effectively rule out the possibility that this initial pool of genes arose by chance. This would, in turn, strengthen the argument for the specific association of the selected core genes with AFB1-induced HCC.

Second, our previous genetic studies based on whole-exome sequencing have revealed that HCC associated with dual exposure to AFB1 and HBV exhibits unique genetic susceptibility characteristics, with molecular mechanisms that are fundamentally different from HCC driven by other etiologies, such as alcohol-related or metabolic-associated fatty liver disease^[2,3]^. The present study, in its analysis of public datasets, did not explicitly mention whether the HBV infection status of the samples was distinguished or subjected to subgroup analysis. Given that HBV infection itself can profoundly influence the host’s molecular pathological environment and potentially crosstalk with AFB1’s carcinogenic pathways, incorporating HBV infection status as a covariate or performing stratified analysis in similar studies could unveil more precise pathogenic networks and core targets of AFB1 in different viral infection contexts. This would not only hold significant epidemiological explanatory value but also enhance the clinical translational potential of the research findings.

Finally, the study employed a “network toxicology” strategy, initially outlining the functional background of the core genes through GO/KEGG enrichment and protein-protein interaction networks. However, traditional enrichment methods based on static gene sets have limitations in capturing the dynamic regulatory relationships and systemic causal chains in complex toxicological responses^[4]^. To further elucidate the carcinogenic mechanisms of AFB1, future research could adopt a network topology analysis framework. This would involve integrating the identified core genes into established AFB1-related oncogenic pathways, such as p53, PI3K-Akt, and Wnt, for topological structure analysis or the construction of pathway reconstruction models. Such an approach could transcend isolated functional annotations, revealing the dynamic regulatory roles of these genes in carcinogenic cascades and the overall characteristics of their interaction networks, thereby deepening the understanding of AFB1-driven carcinogenesis at a systems level.

In summary, the study by Gao *et al*^[1]^, through sophisticated computational analysis, offers insightful findings and identifies important candidate targets for research into the molecular mechanisms of AFB1-induced HCC. We commend the authors for their efforts in addressing this complex scientific problem. To further deepen the understanding of AFB1’s carcinogenic mechanisms and promote its translation into clinical applications, future research should be encouraged to more meticulously consider different subgroups in computational models, particularly the distinct carcinogenic mechanisms in AFB1/HBV dual-exposure HCC. Furthermore, strengthening the integration of computational predictions with subsequent experimental validation is crucial for establishing a more reliable and comprehensive molecular network of AFB1-induced carcinogenesis.

This correspondence adheres to the TITAN Guidelines 2025 for AI reporting standards^[5]^.

## Data Availability

All data generated or analyzed during this study are included in this article. The data are available from the corresponding author upon reasonable request.
